# Identification of QTLs/Defense Genes Effective at Seedling Stage Against Prevailing Races of Wheat Stripe Rust in India

**DOI:** 10.3389/fgene.2020.572975

**Published:** 2020-11-27

**Authors:** Anjan Kumar Pradhan, Sundeep Kumar, Amit Kumar Singh, Neeraj Budhlakoti, Dwijesh C. Mishra, Divya Chauhan, Shikha Mittal, Monendra Grover, Suneel Kumar, Om P. Gangwar, Subodh Kumar, Arun Gupta, Subhash C. Bhardwaj, Anil Rai, Kuldeep Singh

**Affiliations:** ^1^Indian Council of Agricultural Research-National Bureau of Plant Genetic Resources, New Delhi, India; ^2^Indian Council of Agricultural Research-Indian Agricultural Statistics Research Institute, New Delhi, India; ^3^Indian Council of Agricultural Research-Indian Institute of Wheat and Barley Research, Regional Station, Shimla, India; ^4^Indian Council of Agricultural Research-Indian Institute of Wheat and Barley Research, Karnal, India

**Keywords:** stripe rust, wheat, SNP, GWAS, GAPIT

## Abstract

Resistance in modern wheat cultivars for stripe rust is not long lasting due to the narrow genetic base and periodical evolution of new pathogenic races. Though nearly 83 *Yr* genes conferring resistance to stripe rust have been cataloged so far, few of them have been mapped and utilized in breeding programs. Characterization of wheat germplasm for novel sources of resistance and their incorporation into elite cultivars is required to achieve durable resistance and thus to minimize the yield losses. Here, a genome-wide association study (GWAS) was performed on a set of 391 germplasm lines with the aim to identify quantitative trait loci (QTL) using 35K Axiom® array. Phenotypic evaluation disease severity against four stripe rust pathotypes, i.e., 46S119, 110S119, 238S119, and 47S103 (T) at the seedling stage in a greenhouse providing optimal conditions was carried out consecutively for 2 years (2018 and 2019 winter season). We identified, a total of 17 promising QTl which passed FDR criteria. Moreover these 17 QTL identified in the current study were mapped at different genomic locations i.e. 1B, 2A, 2B, 2D, 3A, 3B, 3D, 4B, 5B and 6B. These 17 QTLs identified in the present study might play a key role in marker-assisted breeding for developing stripe rust resistant wheat cultivars.

## Introduction

In wheat, stripe rust caused by *Puccinia striiformis* f. sp. *tritici* (Pst) is the most damaging and widely prevalent disease. It causes significant yield losses in almost every part of the world where cool and humid conditions persist during crop season. In India, stripe (yellow) rust is the major disease in North West Plain Zones (NWPZ) especially sub-mountainous parts of Punjab, Haryana, and Western Uttar Pradesh, the major wheat growing regions of India. Occurrence of stripe rust has also been observed frequently in Jammu & Kashmir, Himachal Pradesh, and tarai regions of Uttarakhand (Chen, 2005; Indu and Saharan, 2011). However, a major outbreak of stripe rust was observed in the North Western Plain Zone (NWPZ) and the Northern Hills Zone (NHZ) of India in 2006 and 2012–13 which caused heavy yield losses (Prashar et al., 2007; Saharan et al., 2013).

Yield losses due to this disease vary from 10 to 40% which depends upon various factors like severity level, susceptibility of cultivars, stage of infection, rate of disease development, and duration of disease. If infection takes place at the seedling stage and the conducive environment persists until maturity, then yield losses may go up to 100% (Afzal et al., 2007).

The presence of large number of pathotypes is the main reason for the epidemic of stripe rust. Out of 140 stripe rust pathotypes known globally, more than 28 pathotypes have been reported in India (Line and Qayoum, 1992; Chen et al., 2010; Bhardwaj et al., 2014; Tomar et al., 2014). As new pathotypes evolve fast, host plants require novel genes to encounter new pathotypes. Although there are more than 83 cataloged *Yr* resistance genes (McIntosh et al., 2017; Li et al., 2020) most of them are all-stage resistance genes with the exception of 22 genes, namely *Yr11-14, Yr16, Yr18/Lr34/Sr57/Pm38/Ltn1, Yr29/Lr46/Sr58/Pm39/Ltn2, Yr30/Lr27/Sr2, Yr36, Yr39, Yr46/Lr67/Sr55/Pm46/Ltn3, Yr52, Yr59, Yr62, Yr68, Yr71, Yr75, Yr77-80*, and *Yr82* (McIntosh et al., 1995; Chen and Kang, 2017; Feng et al., 2018; Nsabiyera et al., 2018; Pakeerathan et al., 2019). In addition, several temporary *Yr* resistance genes and QTL have been identified and reviewed (Rosewarne et al., 2013; Chen and Kang, 2017).

Although rust can be controlled by good agronomical management and fungicides, still cultivation of resistant cultivars is one of the best and most economical options (Wellings, 2011). To develop resistant cultivars at regular intervals, new sources of resistance and new genes/QTLs need to be identified from virgin germplasm lines. GWAS have been found effective for the identification of novel genes/QTLs in germplasm lines, i.e., leaf rust (Kertho et al., 2015), stem rust (Yu et al., 2012; Letta et al., 2013; Laidò et al., 2015), and stripe rust resistance to verify the effect of previously discovered *Yr* genes and QTLs (Tadesse et al., 2013; Zegeye et al., 2014; Naruoka et al., 2015; Bulli et al., 2016; Pasam et al., 2017). Recently, many studies have reported QTLs for stripe rust resistance in wheat using GWAS (Liu et al., 2017a; Muleta et al., 2017a; Yao et al., 2019; Cheng et al., 2020). However, very few GWAS attempts have been made on Indian wheat germplasm lines for identification of stripe rust genes. Most of the landraces used in this study are unexplored and not used in any breeding program. Therefore, the present study was conducted with the aim to identify QTLs/defense genes associated with stripe rust seedling stage resistance through GWAS.

## Materials and Methods

### Plant Material, Inoculation, and Phenotypic Scoring

An association mapping panel of 391 wheat germplasm lines which includes 290 Indian landraces/ indigenous germplasm, 24 exotic, and 77 other germplasm lines including advanced breeding lines and some Indian varieties released during 1960 (Supplementary Table 1). The association mapping panel was evaluated against four virulent and predominant pathotypes of *P. striiformis tritici* in India. The landraces, indigenous and exotic germplasm lines were collected from the National Gene bank of India located at ICAR-NBPGR, New Delhi. The advanced wheat breeding lines used in this analysis were developed at ICAR-Indian Institute of Wheat and Barley Research (IIWBR) Karnal. All the 391 wheat germplasm lines were evaluated at the seedling stage using a mixture of Pst pathotypes prevalent in India over 2 years (2018 and 2019 winter season) under greenhouse conditions at IIWBR Regional Station, Shimla. Reference lines known to possess specific *Yr* gene/s commonly occurring in wheat were used to confirm the purity of pathotypes (Bhardwaj et al., 2012). A mixture of fresh garden soil and FYM (1:1 ratio), autoclaved at 60°C for 4 h was used for growing wheat plants in plastic pots and aluminum bread pans/trays. In an aluminum bread pan tray, 4–5 seeds of each line were clump planted. Each tray contained 18 lines and a susceptible check (A-9-30-1). In plastic pots 10–12 seedlings were raised. After seeding, the trays/pots were kept in the greenhouse at 22 ± 2°C with proper labels and provided with optimum conditions to ensure normal germination and growth of the seedling.

After a week, wheat seedlings were inoculated with each pathotype separately by atomizing the uredosporic inoculum suspended in non-phytotoxic isoparaffinic oil (commercially known as Soltrol 170 produced by Chevron Phillips Chemicals Asia Pvt. Ltd., Singapore). The inoculated plants were fine sprayed with water and incubated for 24 h in water saturated glass chambers. The plants were then transferred to the greenhouse and dusted with elemental dust powder of Sulfur to avoid the occurrence of powdery mildew infection. All the optimum greenhouse conditions for good plant growth and proper stripe rust infection were provided. The greenhouse temperature was maintained at 15 ± 1°C. The response of host-pathogen interactions was recorded in the form of infection type after 16–18 days of inoculation following Nayar et al. (1997). Disease severity was recorded on 10 plants for each germplasm line. Wheat-rust infection responses (low or high) were recorded on experimental material and differentials (to ascertain the purity of pathotypes) by following McNeal et al. (1971) using a 0–9 scale where, 0 indicates Immune (no observed visible infection), 1 means highly resistant (necrotic/chlorotic flecks appears without sporulation), 2 means resistant (there are necrotic/chlorotic stripes without sporulation) 3 shows moderately resistant (trace sporulation, necrotic/chlorotic stripes are there), 4 represents moderately resistant (light sporulation, necrotic/chlorotic stripes appears), 5 suggests moderately susceptible (intermediate sporulation, necrotic/chlorotic stripes appears on leaves), 6 specify highly moderate(moderate sporulation, necrotic/chlorotic stripes were termed as resistant), whereas 7 means moderately susceptible (abundant sporulation, necrotic/chlorotic stripes can be observed on the leaves), 8 means susceptible (abundant sporulation, with chlorosis), while 9 is highly susceptible (plant shows abundant sporulation, without chlorosis, were categorized as susceptible).

### Phenotypic Data Analysis

To determine genotypic and year variances among pathotypes, Analysis of variance (ANOVA) was performed using SAS v9.3 (software). Frequency distribution of genotypes for different pathotypes under study has also been generated showing the performance of phenotypes. Heritability of the pathotypes infection was estimated using the restricted maximum likelihood (REML) method. For the analysis of 2-years data, pathotype mean, variance, standard deviation, and ranges of each germplasm line was calculated.

### Genotyping

Leaves from 15-days old seedlings were collected and standard CTAB protocol was followed for genomic DNA extraction (Doyle, 1990). A total of 391 germplasm lines were genotyped using the 35K Axiom® array (Affymetrix product ID 550524) for wheat having 35,143 SNPs. SNPs having low-quality clustering and minor allele frequency (MAF ≤ 10%), across all the genotypes were excluded from the analysis. To assign an exact physical location to each SNP on wheat chromosomes, SNP probe sequences were subjected to BLAST against wheat reference genome RefSeq v1.0 (https://wheat-urgi.versailles.inra.fr/Seq-Repository/Assemblies) following default parameters. A total of 19,090 polymorphic SNPs were assigned to an exact physical location on the wheat genome and used for further downstream analysis.

### Analyses of Molecular Diversity and Population Structure

A set of 525 random SNP markers distributed across the 21 wheat chromosomes (25 markers per chromosome) were used to determine the population structure. The STRUCTURE v2.3.4 software based on admixture model with correlated allele frequency was applied to categorize sub-population in the current germplasm lines (Pritchard et al., 2000). For Structure, parameters like 20,000 iterations and 50,000 Monte Carlo Markov Chain (MCMC) replicates were set to determine K values in the range of 1–10. For each K value, ten independent structure runs were carried out and further the results were exported to Structure Harvester (http://taylor0.biology.ucla.edu/structureHarvester/) software for determining the most likely number of subpopulations in germplasm lines (Evanno et al., 2005). Further, fixation index (Fst) for subpopulations was estimated from various STRUCTURE runs. Principal components (PC) were also inferred using the Genomic Association and Prediction Integrated Tool (GAPIT) R package to further analyze population sub-structuring and a comparison was made from the results analyzed with STRUCTURE. To further determine the genetic structure of the lines, cluster analysis based on the neighbor joining (NJ) tree algorithm according to shared-allele distance was also performed in TASSELv5.0. The branching pattern in the NJ tree was assessed based on bootstrapping over loci with 1,000 replications, while the consensus bootstrap value was displayed with the help of ITOL program v5 (https://itol.embl.de/).

### Linkage Disequilibrium Analysis

Linkage disequilibrium (LD) based on pairwise measures between SNP markers were estimated using TASSELv5.0 (Bradbury et al., 2007). LD can be estimated as squared allele frequency correlation (R^2^) between pairs of intra-chromosomal SNPs with known chromosomal position. The background LD in the wheat AM panel was calculated to identify critical distance for LD decay. The average pattern of genome-wide LD decay over physical distance was determined by constructing a scatterplot of R^2^ values against the corresponding physical distance among markers. Further, the extent of LD decay was also estimated using the Locally Weighted Scatter-plot Smoother (LOESS) model (Cleveland, 1979). The critical R^2^ value that shows the area beyond which LD is due to true physical linkage was determined using 95th percentile of the square root of transformed R^2^ data of unlinked markers (Breseghello and Sorrells, 2005). Further, the intersection of LD decay curve was observed at R^2^ = 0.156 and at 2.5 Mb distance. Therefore, all the significantly associated SNPs (clustered SNPs) falling within this distance were designated as single QTLs.

### Genome-Wide Association Analysis

GWAS was conducted using a panel of 19,090 high quality polymorphic SNP markers and disease severity data against four different pathotypes 47S103, 238S119, 46S119, and 110S119 at seedling stage. This analysis was done using the phenotyping data from both the year (2018 and 2019) separately as well as for pooled data. We have presented the GWAS results for the marker using pooled data, however we have also highlighted the markers which were consistent in both years. Marker-trait associations (MTAs) were identified using the compressed mixed linear model (CMLM) (Yu et al., 2006; Zhang et al., 2010) implemented in GAPIT R package (Lipka et al., 2012). CMLM uses the additional information like Principal component (usually three components) and Kinship of population (K), hence it is also called PC3 + K CMLM model. CMLM is compressed form of mixed linear model. The general equation of MLM can be written as follows

$$\begin{array}{l} y=X\beta +Zu+e \end{array}$$

Where, y = observed phenotypic vector, β = vector of marker fixed effects; u = vector of random additive genetic effects from individual lines; e = vector of residuals; X and Z are known design matrices.

*P*-value and R^2^ were used as parameter to identify significant marker-trait associations (MTAs). Significant MTAs were identified at the threshold of *P* < 0.001. In order to show the distribution of SNPs over the chromosome, Manhattan plots have also been generated.

### Identification of Putative Candidate Gene and Their Annotation

Candidate genes and their corresponding molecular functions were retrieved from the wheat genome assembly IWGSC Ref-Sequence v1.1 using BLASTN function of BLAST program with default parameters. Associated SNPs were extracted along with their annotations for transcript located within the distance. The identified putative candidate genes were further validated using stripe rust disease resistance data from Sequence Read Archive (SRA) NCBI database (Bio Project—PRJNA613349).

## Results

### Analysis of Phenotypic Variation for Disease Severity at Seedling Stage

**Figure 1:**
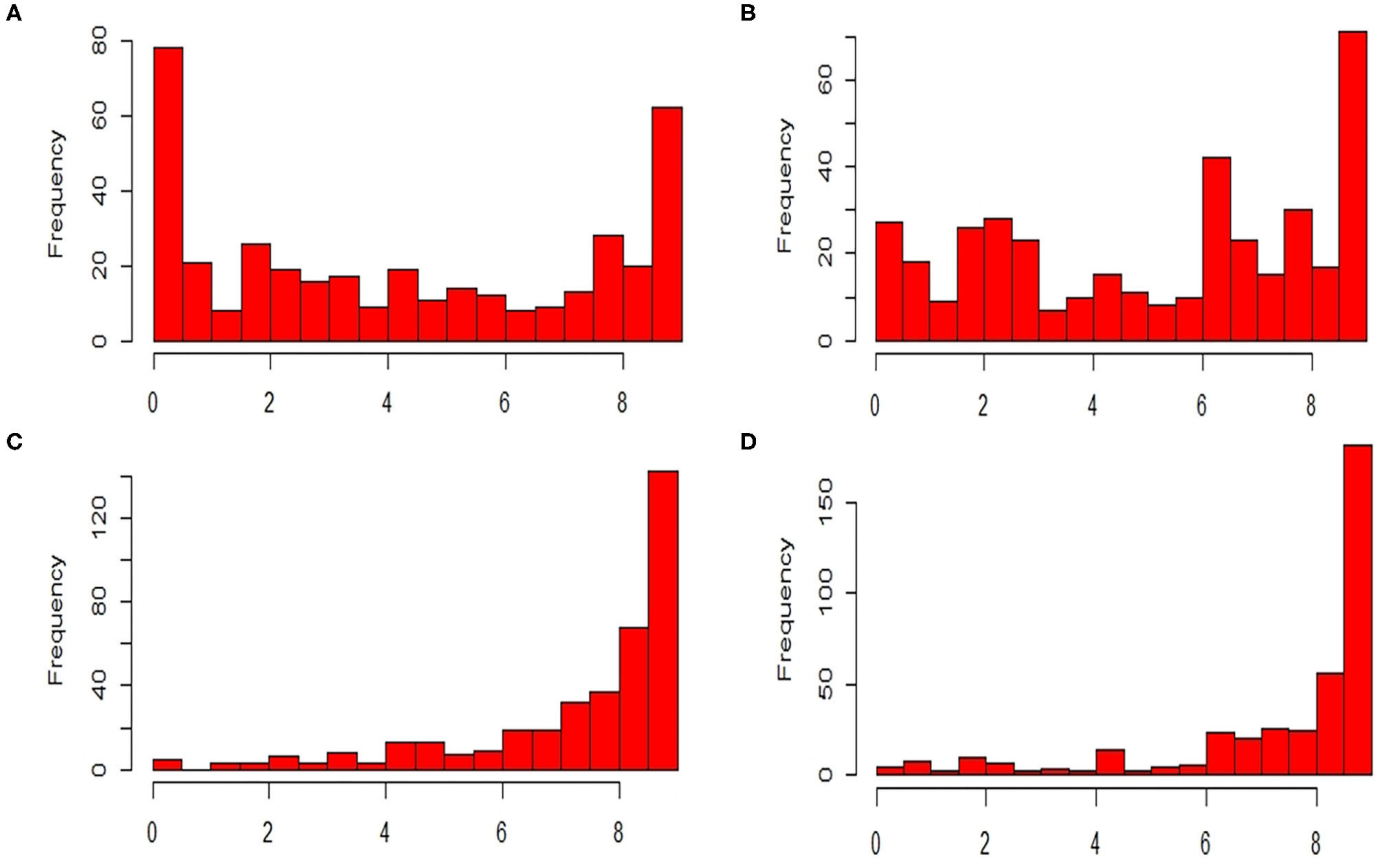
Frequency distributions of stripe rust resistant pathotypes **(A)** 110S119, **(B)** 238S119, **(C)** 46S119, and **(D)** 47S103.

**Figure 2:**
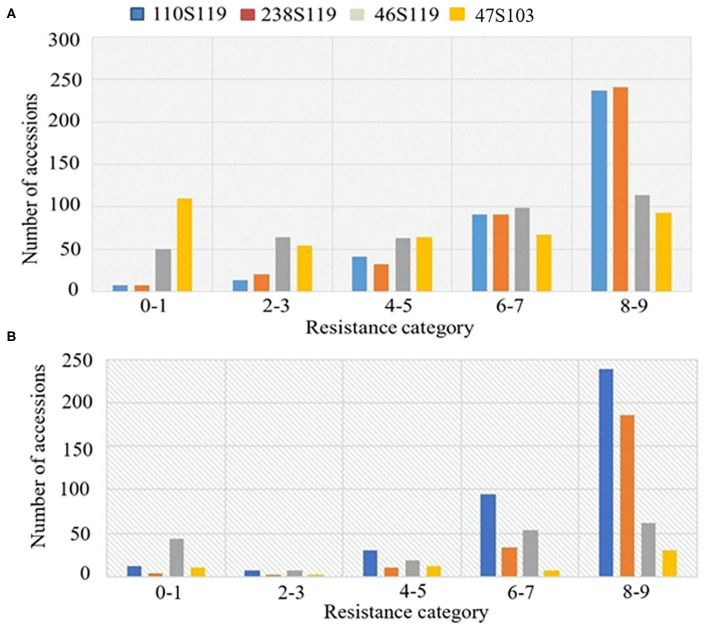
Frequency distributions of stripe rust resistance types produced by seedlings of the bread wheat genotypes **(A)** 2018, **(B)** 2019.

The ANOVA results revealed highly significant variation (*P* < 0.0001) for germplasm lines and pathotypes (Table 1) while the genotype × year interaction effect was found to be non-significant. Further, the frequency distribution of the infection types (ITs) produced by the four different Pst races (a) 110S119, (b) 238S119, (c) 46S119, and (d) 47S103 in association mapping panel is shown in Figures 1A–D. Among 391 germplasm lines, 35 (10%), 40 (11%), 144 (37%), and 189 (49%) showed (Supplementary Table 2) seedling resistance to races 110S119, 238S119, 46S119, and 47S103, respectively in 1st year and 33 (9%), 37 (10%), 144 (37%), 181 (47%), respectively in 2nd year (Figure 2B). A higher percentage of germplasm lines were susceptible (IT = 8–9) to races 110S119 (60%), 238S119 (61%), 46S119 (29%), and 47S103 (23%) (Figure 2A) in first year as compared to the second year 110S119 (61%), 238S119 (47%), 46S119 (15%), and 47S103 (7%). Further, the heritability (*h*^2^) value for stripe rust infection type (IT) was 0.58 (Table 2).

**Table 1:** Analysis of variance (ANOVA) for reaction to stripe rust bread wheat.

**Stage**	**Sources**	***df***	***F***	**Sig**
	Genotypes (G)	390	3.07	^***^
Seedling	Year (Yr)	1	9.44	0.0021
	Race	3	240.67	^***^
	G*Yr interaction	390	0.51	1.0000

*** *Significant difference at P < 0.0001*.

**Table 2:** Descriptive statistics and heritability of races.

**Races**	**Range**	**Mean ± SE**	**Variance**	***SD***	**Heritability**
47S103	0.0-9	4.58 ± 0.16	10.81	3.28	0.58
46S119	0.0-9	5.24 ± 0.15	9.6	3.09	
110S119	0.0-9	7.48 ± 0.09	3.78	1.94	
238S119	0.0-9	7.55 ± 0.1	4.41	2.1	

### Marker Coverage and Population Structure Analysis

A total of 19,090 SNPs assigned to an exact physical location on the wheat genome were used for association mapping. Out of 19,090 SNPs, 6,083 were mapped on A sub-genome, 7,253 on B sub-genome and 5,754 on D sub-genome. The number of SNPs on individual chromosomes ranged from 378 on 4D to 1,315 on 2B. Chromosome level distribution of SNPs represented that A sub-genome possesses maximum SNPs on 2A (1,079), followed by 1A (1,053) and 7A (953). Whereas, in the B sub-genome, the maximum SNPs were on 2B (1,315), followed by 5B (1,257) and 1B (1,159). While, in the case of D sub-genome, maximum SNPs were on 2D (1,176) followed by 1D (1,039) and 5D (897) (Table 3).

**Table 3:** Genetic clusters and their member genotypes, proportion of membership, expected heterozygosity, and the mean values of Fst observed from structure analysis of 391 Stripe rust resistance wheat genotypes.

**Cluster**	**Genotypes**	**% Membership**	**Expected heterozygosity**	**Mean fixation index**
1	205	0.496	0.3438	0.2958
2	102	0.240	0.1809	0.6170
3	10	0.070	0.0917	0.8336
4	74	0.194	0.3358	0.2385

**Figure 3:**
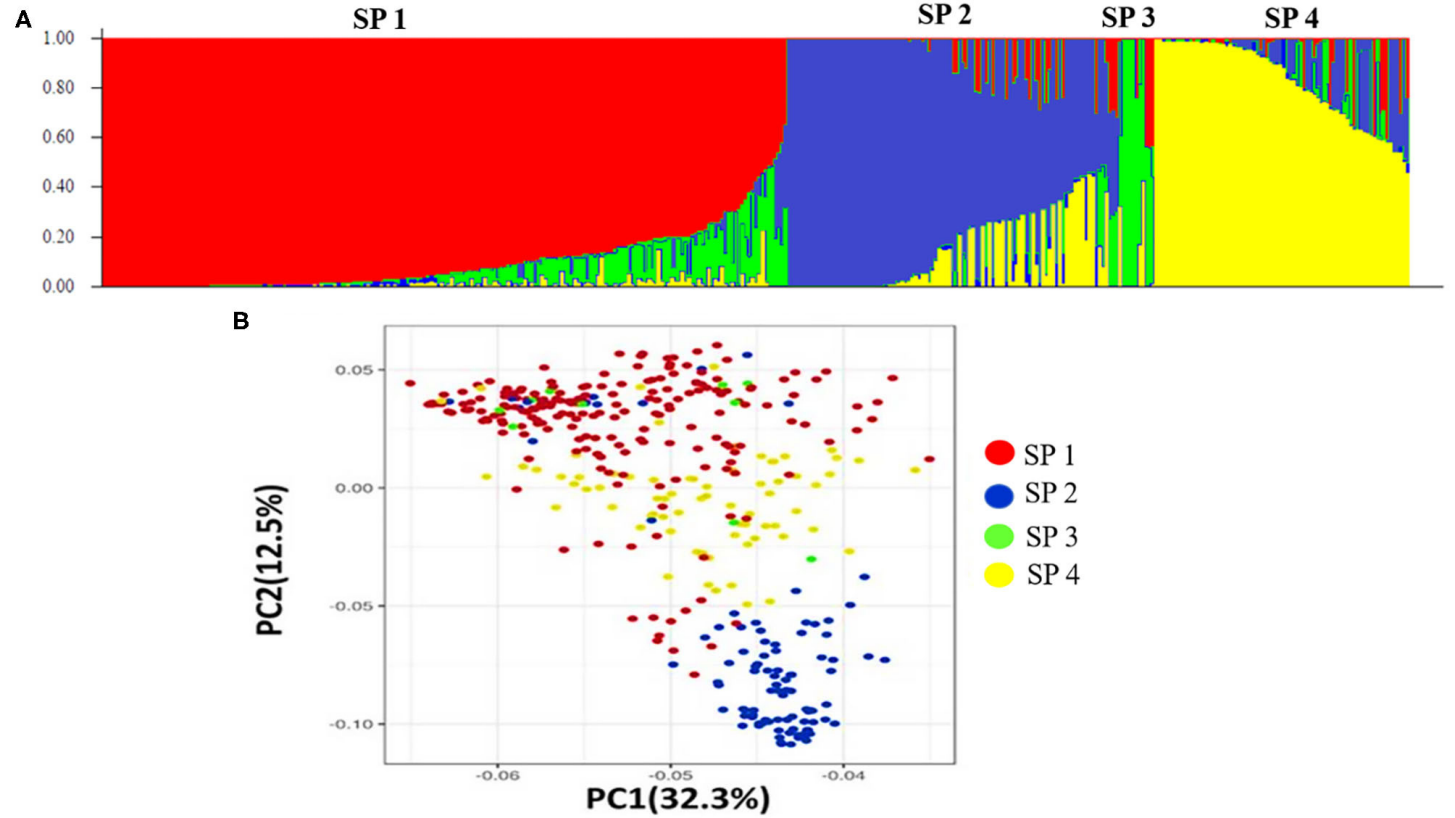
Population structure of the 391 stripe rust germplasm lines combined with markers. **(A)** Population structure of the bread wheat among 19,090 SNP polymorphism markers, **(B)** Principal component analysis.

Population structure analysis categorizes the 391 genotypes into four sub-populations (SP). Containing 205, 102, 10, and 74 genotypes, respectively. Sub-populations 1 (SP 1) was the largest among the four sub-populations, having 52.2% of the genotypes from the association mapping panel followed by sub-population 2 (SP 2), sub-population 4 (SP 4), and the smallest one sub-population 3 (SP 3), i.e., 2.5% (Table 4). Sub-populations 1 and 4 revealed the highest level of heterozygosity, i.e., 0.3438 and 0.3358, respectively. Individuals of each subpopulation were further categorized as pure and admixtures in type based on membership proportion. Genotypes that had a membership proportion of ≥0.8 were considered pure and genotypes <0.8 were considered admixtures. Based on this criterion, composition of four sub-populations was as follows; SP 1–60% pure and 40% admixtures; SP 2–36% pure and 64% admixtures; SP 3–40% pure and 60% admixtures and SP 4–37% pure and 63% admixtures (Figure 3A). Out of four subpopulations, SP 1 consists of a higher proportion of susceptible germplasm lines than others. In SP 1, most of the germplasm lines were susceptible to pathotype 238S119 and highly resistant to pathotype 46S119. On the other hand, in SP 2, most of the germplasm lines were susceptible to pathotype 110S119 and highly resistant to stripe rust pathotype 46S119. For the SP 4, maximum genotypes were resistant against 47S103 followed by 46S119, while susceptibility was for the 238S119 followed by 110S119.

**Table 4:** Distribution of 19,090 SNPs in 21 chromosomes identified LD in 391 resistant of Stripe rust bread wheat genotypes.

**Chr**	**Size (Mb)**	**No. of polymorphic SNP**	**Average number of SNPs per Mb**	**Chromosome (LD)**	**No. of marker pairs in perfect LD (R^**2**^ = 1)**
1A	594.1	1,053	1.77	0.212	1,803
1B	689.85	1,159	1.68	0.303	3,496
1D	495.45	1,039	2.10	0.325	4,523
2A	780.8	1,079	1.38	0.187	1,377
2B	801.26	1,315	1.64	0.160	746
2D	651.85	1,176	1.80	0.205	1,647
3A	750.84	783	1.04	0.122	244
3B	830.83	1,145	1.38	0.158	361
3D	615.55	692	1.12	0.105	118
4A	744.59	652	0.88	0.119	224
4B	673.62	550	0.82	0.108	191
4D	509.86	378	0.74	0.088	89
5A	709.77	828	1.17	0.126	298
5B	713.15	1,257	1.76	0.185	1,340
5D	566.08	897	1.58	0.152	776
6A	618.08	735	1.19	0.154	427
6B	720.99	975	1.35	0.148	300
6D	473.59	725	1.53	0.111	158
7A	736.71	953	1.29	0.129	352
7B	750.62	852	1.14	0.126	200
7D	638.69	847	1.33	0.102	172

**Figure 4:**
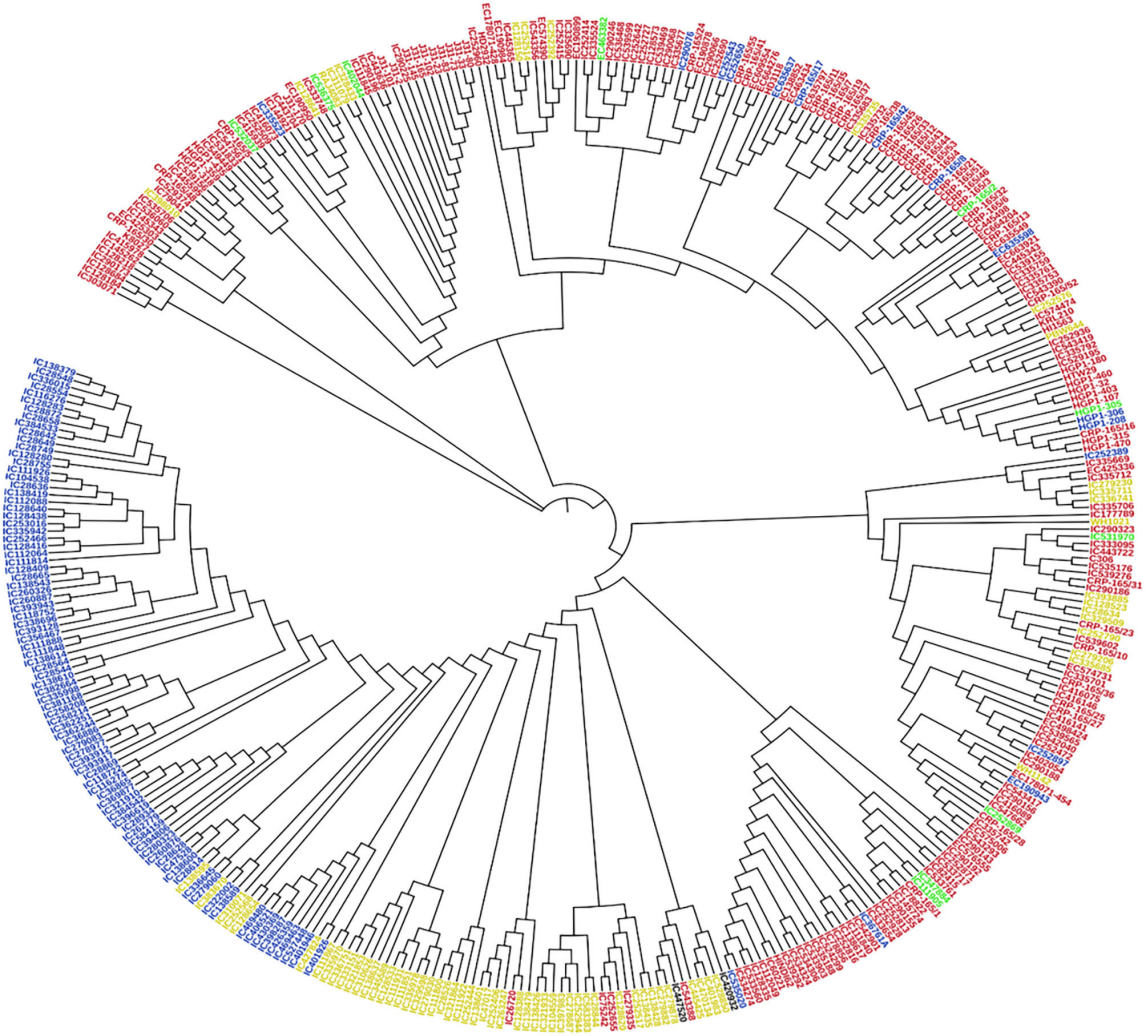
Cluster analysis was based on the neighbor-joining (NJ) algorithm. Genotypes were divided into four subpopulations using STRUCTURE (*K* = 4). Red, Blue, Green, and Yellow color represents SP 1, SP 2, SP 3, and SP 4, respectively.

Principal component analysis (PCA) was also performed using 391 genotypes to estimate population structure including 1st three PCs for further downstream analysis. PC1 and PC2 have explained 32.3 and 12.5% of the genetic variance, respectively. The PC analysis scatter plot (Figure 3B) also confirmed the results of population structure analysis as it showed that 1st and 2nd PCs were composed mainly by four sub-populations. Moreover, cluster analysis was carried out based on the neighbor-joining (NJ) algorithm that revealed four clusters in an association panel. Here, the neighbor-joining (NJ) tree of the stripe rust resistance wheat lines was evaluated by shared-allele genetic distance using high-density SNP markers (Figure 4).

### Linkage Disequilibrium (LD)

**Figure 5:**
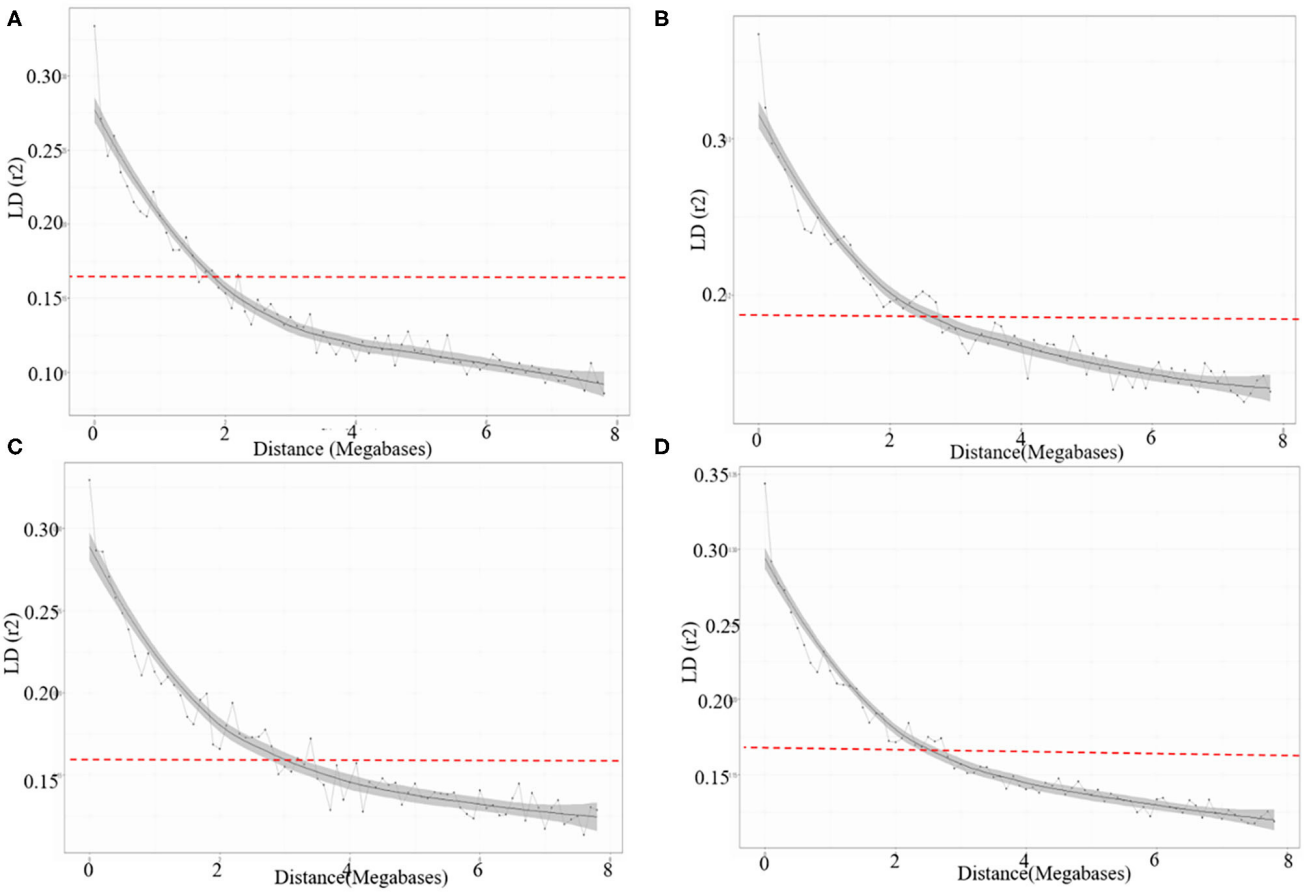
LD decay across the **(A)** A, **(B)** B, **(C)** D sub-genomes, and **(D)** whole genome of wheat. The values on the Y-axis represent the squared correlation coefficient (R^2^) and the values at X-axis represent physical distance (Mb).

The LD for each sub-genome, i.e., A, B, and D genome was estimated from all pairs of SNPs present over there. Individually the average R^2^ of genome wide LD was 0.14 for A sub-genome, 0.16 for B sub-genome, and 0.15 for D sub-genome. SNP markers, whose map positions were assigned, were further used to estimate intra-chromosomal LD. A total of 31.46% intra-chromosomal pairs loci were in significant LD (i.e., R^2^ of 0.2), while 18,842 SNP pairs were in a perfect LD (i.e., R^2^ = 1). The decay of LD across the genome is an important parameter that determines the number of significant markers required for performing GWAS analysis. The extent of LD distribution was graphically demonstrated by plotting intra-chromosomal R^2^ values for loci against their physical distance and a second-degree LOESS curve was also fitted for further exploration. The background LD in the analyzed AM panel was equal to 0.156 and taken as the threshold cut-off for estimating LD decay. In the selected wheat panel LD decayed the fastest in the A sub-genome comparison to B and D. In the A sub genome R^2^ value for the marker pairs reached 0.156 (used as a threshold) at 1.9 Mb as compared to 2.3 Mb in B and 2.9 Mb in D sub-genomes (Figures 5A–D).

### Genome-Wide Association Analysis

The association of SNP markers with stripe rust resistance against four different pathotypes at seedling stages were determined by CMLM analysis using kinship (K) matrix and population structure (Q matrix) as a covariate. A total of forty QTLs (51 MTAs) in 19 genomic regions (1A, 1B, 2A, 2B, 2D, 3A, 3B, 3D, 4A, 4B, 4D, 5A, 5B, 5D, 6A, 6B, 6D, 7A, and 7D) were linked with resistance to four pathotypes (at *P* < 0.001, and out of these 40 QTLs, 17 QTLs were significant at FDR adjusted *P* < 0.30).

**Figure 6:**
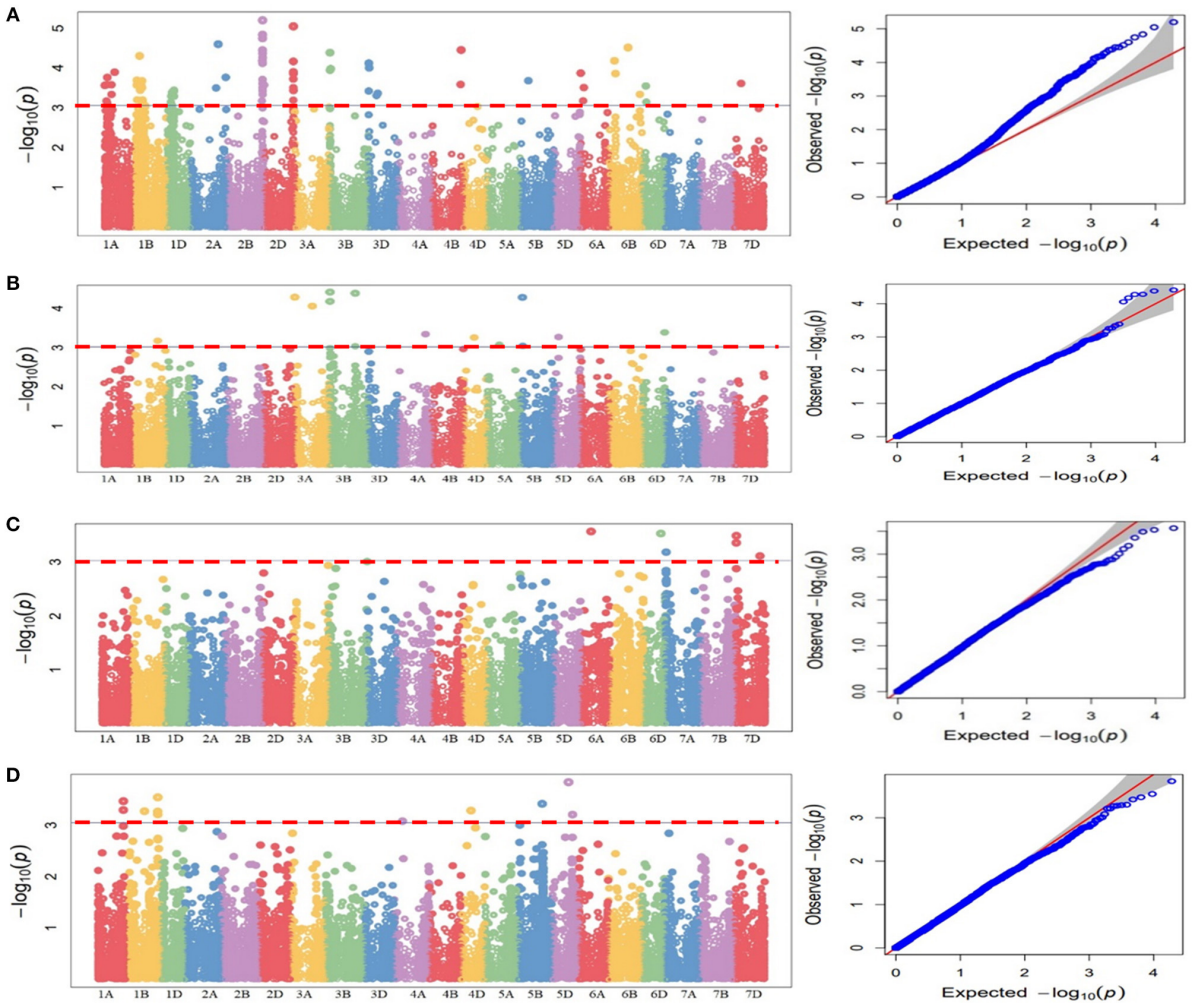
Manhattan plots for statistical significance *P*-values across 21 wheat chromosomes for SNP markers associated with stripe rust four pathotypes. **(A)** 47S103, **(B)** 46S119, **(C)** 110S119, **(D)** 238S119 and Quantile-Quantile plots indicating the normality of data four pathotypes. Horizontal line represents significant MTAs at threshold of *P* < 0.001.

These QTLs were distributed as follows; 12 QTLs for 47S103, 12 QTLs for 46S119, 7 QTLs for 110S119, and 9 QTLs for 238S119. Coefficient of determination (R^2^) for each QTL was also determined. Phenotypic variance ranged from 6.6 to 28.8%. Ability of the GWAS model was tested using Manhattan plots and quantile-quantile-plots (Q-Q plots) between observed and expected *P*-values of association, that revealed a good fitting for the model with population structure and kinship (Table 5, Figures 6A–D), i.e., (a) 47S103, (b) 46S119, (c) 110S119, and (d) 238S119.

**Table 5:** Quantitative trait loci for stripe rust resistance identified at the seedling stage using pooled data.

**Pathotype**	**QTL**	**SNP**	**Chr**	**Position (Mb)**	**Allele**	**MAF**	***P*-value**	**FDR *P*-value**	**R^**2**^ (%)**	**Allelic Effect**
**47S103**	*Qyr.stripe-1BS*	AX-94839474*	1BS	92.87	T/C	0.194	4.95E-05	0.084702^#^	27.7	1.84
	*Qyr.stripe-2AL*	AX-95174135	2AL	591.62	C/T	0.363	2.52E-05	0.081406^#^	28.1	−1.05
	*Qyr.stripe-2BL.1*	AX-94848091	2BL	756.70	C/T	0.232	6.53E-05	0.081406^#^	27.6	1.02
	*Qyr.stripe-2BL.2*	AX-94490490	2BL	759.82	C/T	0.378	3.54E-05	0.081406^#^	27.9	−1.03
		AX-95175933	2BL	760.12	C/T	0.203	1.46E-05	0.081406^#^	28.3	−2
		AX-94608940	2BL	760.26	A/G	0.498	7.54E-05	0.081406^#^	27.5	1.12
		AX-95142963	2BL	760.89	A/C	0.387	1.79E-05	0.081406^#^	28.2	−1.07
		AX-95238626*	2BL	760.95	G/A	0.195	4.26E-05	0.084702^#^	27.8	1.86
		AX-94497849	2BL	761.98	C/T	0.31	5.47E-05	0.084702^#^	27.7	1.91
		AX-94868242*	2BL	761.99	A/C	0.475	6.33E-06	0.084702^#^	28.8	−1.03
	*Qyr.stripe-2DL.1*	AX-95007159	2DL	619.60	A/G	0.441	6.80E-05	0.081406^#^	27.6	1.16
	*Qyr.stripe-2DL.2*	AX-95166280	2DL	621.02	G/A	0.194	8.98E-06	0.084702^#^	28.6	1.97
	*Qyr.stripe-3B*	AX-94877000*	3B-	3.24	A/T	0.427	4.10E-05	0.081406^#^	27.8	−0.98
	*Qyr.stripe-3DS.1*	AX-95098367	3DS	3.98	A/G	0.256	7.54E-05	0.084702^#^	27.5	−0.96
	*Qyr.stripe-3DS.2*	AX-94417619	3DS	12.45	C/G	0.435	9.85E-05	0.104201^#^	27.4	0.94
	*Qyr.stripe-4BL*	AX-94853722	4BL	623.07	C/A	0.422	3.52E-05	0.081406^#^	27.9	−1.39
	*Qyr.stripe-6BL.1*	AX-94735973	6BL	117.99	C/T	0.413	6.56E-05	0.081406^#^	27.6	1.62
	*Qyr.stripe-6BL.2*	AX-94382687	6BL	408.65	G/A	0.162	3.05E-05	0.084702^#^	28	1.34
**46S119**	*Qyr.stripe-1BL*	AX-94482117	1BL	486.48	T/G	0.134	6.69E-04	0.97075	11.8	1.14
	*Qyr.stripe-3AL*	AX-94419836*	3AL	374.73	G/T	0.182	8.77E-05	0.250823^#^	13	1.02
	*Qyr.stripe-3AS*	AX-95193648	3AS	0.56	A/G	0.172	5.19E-05	0.278927^#^	13.3	−1.58
	*Qyr.stripe-3B.1*	AX-94486149	3B-	3.23	G/A	0.221	6.66E-05	0.250823^#^	13.2	1.24
		AX-94904447	3B-	3.24	G/T	0.197	3.86E-05	0.250823^#^	13.5	−1.53
	*Qyr.stripe-3B.2*	AX-94702815*	3B-	542.74	A/T	0.199	4.12E-05	0.254159^#^	13.5	−1.94
		AX-94701179*	3B-	542.90	A/G	0.373	9.24E-04	0.97075	11.7	−0.86
	*Qyr.stripe-4AL*	AX-95630255	4AL	602.25	G/A	0.199	4.55E-04	0.97075	12.1	−0.95
	*Qyr.stripe-4DL*	AX-94541083*	4DL	226.26	T/C	0.142	5.52E-04	0.97075	11.9	1.47
	*Qyr.stripe-5AS*	AX-94797468	5AS	253.60	C/T	0.454	8.48E-04	0.97075	11.7	−0.82
	*Qyr.stripe-5BS.1*	AX-94706157	5BS	38.36	C/A	0.486	5.26E-05	0.250823^#^	13.3	0.85
	*Qyr.stripe-5BS.2*	AX-95145565*	5BS	45.24	C/T	0.154	9.01E-04	0.97075	11.7	−1.22
	*Qyr.stripe-5DS*	AX-94429414	5DS	98.26	C/T	0.019	5.36E-04	0.97075	12	−3.97
	*Qyr.stripe-6DL*	AX-94859969	6DL	462.86	G/A	0.306	4.11E-04	0.97075	12.1	0.85
**110S119**	*Qyr.stripe-3B*	AX-94883935	3B-	816.28	A/C	0.291	9.76E-04	1	6.6	0.59
	*Qyr.stripe-6AS*	AX-94989376	6AS	221.88	T/G	0.177	2.70E-04	1	7.4	−1.01
	*Qyr.stripe-6DL*	AX-94633926	6DL	369.66	C/G	0.279	2.95E-04	1	7.4	−0.48
	*Qyr.stripe-7AS*	AX-94844913	7AS	8.78	A/G	0.5	6.54E-04	1	6.9	0.4
	*Qyr.stripe-7DL*	AX-95227592	7DL	518.05	A/G	0.205	7.70E-04	1	6.8	0.48
	*Qyr.stripe-7DS.1*	AX-94943939	7DS	14.17	A/G	0.234	4.39E-04	1	7.1	0.5
	*Qyr.stripe-7DS.2*	AX-94878859	7DS	16.70	A/G	0.393	3.24E-04	1	7.3	0.51
**238S119**	*Qyr.stripe-1AL*	AX-95153545*	1AL	541.98	G/A	0.195	5.01E-04	0.993302	8.2	0.64
		AX-94381637	1AL	542.87	C/T	0.14	3.37E-04	0.993302	8.4	−0.82
	*Qyr.stripe-1BL.1*	AX-94717933	1BL	326.02	C/T	0.414	5.28E-04	0.993302	8.1	0.75
	*Qyr.stripe-1BL.2*	AX-95243592	1BL	606.84	G/T	0.152	5.36E-04	0.993302	8.1	−0.7
		AX-95229302	1BL	608.47	C/T	0.171	2.83E-04	0.993302	8.5	−0.69
		AX-94850928	1BL	608.53	C/T	0.168	6.11E-04	0.993302	8.1	−0.66
	*Qyr.stripe-4AS*	AX-94398581	4AS	176.88	C/T	0.229	8.25E-04	0.993302	7.9	−0.55
	*Qyr.stripe-4DL*	AX-94948233	4DL	211.03	T/C	0.371	5.13E-04	0.993302	8.2	−0.63
	*Qyr.stripe-5BL*	AX-95258852	5BL	499.12	T/C	0.422	3.79E-04	0.993302	8.3	−0.82
	*Qyr.stripe-5BS*	AX-94809513	5BS	26.45	T/C	0.317	9.81E-04	0.993302	7.8	0.69
	*Qyr.stripe-5DL.1*	AX-95244380*	5DL	344.50	C/G	0.146	1.44E-04	0.993302	8.9	−0.8
	*Qyr.stripe-5DL.2*	AX-95202253	5DL	428.83	G/A	0.114	6.15E-04	0.993302	8.1	−0.97

Here * denotes MTAs which were also found to be significant to both year (i.e. 2018 and 2019) association mapping results and # represents FDR adjusted p < 0.30.

Twelve QTLs for 47S103 were mapped on 8 different chromosomes (i.e., 1B, 2A, 2B, 2D, 3B, 3D, 4B, and 6B) and most of them were on B sub-genome. Only 1 QTL was mapped on the A sub-genome (*Qyr.stripe-2AL* with *p*-value 2.52E-05 and R^2^ = 0.28). Seven QTLs were mapped on B sub genome, out of which 2 QTLs, i.e., *Qyr.stripe-2BL.1* and *Qyr.stripe-2BL.2*, were found on the long arm of the chromosome 2B between 760 and 762 Mb (2.17 Mb region). Four QTLs were mapped on D sub-genome (*Qyr.stripe-2DL.1, Qyr.stripe-2DL.2, Qyr.stripe-3DS.1, Qyr.stripe-3DS.2*) at different positions.

For pathotype 46S119, a total of 12 QTLs were identified which mapped on nine different chromosomes (1B, 3A, 3B, 4A, 4D, 5A, 5B, 5D, and 6D), most of them were mapped on B sub-genome. In these QTLs, R^2^ ranged from 11.7 to 13.5%. Four QTLs were mapped on A sub-genome (i.e., *Qyr.stripe-3AL, Qyr.stripe-3AS, Qyr.stripe-4AL, Qyr.stripe-5AS*) at various locations. Further, 5 QTLs were mapped on B sub-genome, 2 of them, i.e., *Qyr.stripe-3B.1, Qyr.stripe-3B.2* were found on 3B at 3 Mb and 542 Mb location as a close neighbor at 0.01 and 0.16 Mb apart, respectively. Other 3 QTLs were mapped on D sub-genome (*Qyr.stripe-4DL, Qyr.stripe-5DS, Qyr.stripe-6DL*).

In the case of pathotype 110S119, 7 significant QTLs were mapped on five different chromosomes (3B, 6A, 6D, 7A, and 7D). The highest number of QTLs were mapped on D sub-genome, i.e., 4 QTLs (*Qyr.stripe-6DL, Qyr.stripe-7DL, Qyr.stripe-7DS.1, Qyr.stripe-7DS.2*) and 2 QTLs were mapped on A sub-genome (*Qyr.stripe-6AS, Qyr.stripe-7AS*), whereas only 1 QTL was found on B sub-genome (*Qyr.stripe-3B* with *p*-value 9.76E-04 and R^2^ = 0.07). Further for the pathotype 238S119, 9 QTLs were identified as significant. These QTLs were found on 6 different chromosomes at various locations (i.e., 1A, 4A, 1B, 5B, 4D, and 5D). Maximum number, i.e., 4 QTLs were found to be associated with B sub-genome (*Qyr.stripe-1BL.1, Qyr.stripe-1BL.2, Qyr.stripe-5BL, Qyr.stripe-5BS*) followed by D (*Qyr.stripe-4DL, Qyr.stripe-5DL.1, Qyr.stripe-5DL.2*) and A sub-genome (*Qyr.stripe-1AL, Qyr.stripe-4AS*).

Furthermore, in order to identify consistent MTAs across the years, GWAS was separately performed for 2018 and 2019. The results are presented in Supplementary Tables 3, 4. A total of 9 QTLs were consistently present in 2018 and 2019, i.e., in agreement with the results of the pooled analysis. These include 3 QTLs for 47S103 (1BS: *Qyr.stripe-1BS*, 2BL: *Qyr.stripe-2BL.2* and 3B: *Qyr.stripe-3B*), 4 QTLs for 46S119 (3AL: *Qyr.stripe-3AL*, 3B: *Qyr.stripe-3B.2*, 4DL: *Qyr.stripe-4DL* and 5BS: *Qyr.stripe-5BS.2*), and 2 QTLs for 238S119 (1AL: *Qyr.stripe-1AL* and 5DL: *Qyr.stripe-5DL.1*).

### Identification of Putative Candidate Genes

Candidate genes for pathotypes 47S103, 46S1119, 110S119, and 238S119 were identified by mapping the markers on wheat genome assembly. Seven prominent markers (47S103-2BL, i.e., for pathotype 47S103 on long arm of B sub-genome) mapped to 2.17 Mb interval ranging from 759.82 Mb (AX-94490490) to 761.99 Mb (AX-94868242) on chromosome 2BL. This interval contains 25 genes, of which 5 had a high enrichment score. Similarly, 238S119-1BL mapped to 1.69 Mb interval ranging from 606.84 Mb (AX-95243592) to 608.53 Mb (AX-94850928) on chromosome 1BL. This interval contains seven genes, of which two were had a high enrichment score. Interestingly, the two markers, i.e., AX-94877000 and AX-94904447 associated with 47S103 and 46S119 pathotypes, respectively were linked with a common gene (*TraesCS3B02G005900*) at 3.24 Mb on chromosome 3B. For pathotypes 46S119, 110S119, and 238S110 three of these genes, *TraesCS6D02G384800, TraesCS7A02G021700*, and *TraesCS1B02G376000* mapped on chromosome 6DL, 7AS, and 1BL, respectively. These genes were annotated as leucine-rich repeat receptor-like protein kinases (LRR) and serine-threonine/tyrosine-protein kinase (STPK) which have key roles in pathogen recognition and disease resistance.

The annotation of associated SNPs revealed potential candidate genes (Supplementary Table 5 and Table 6). Maximum candidate genes were found for pathotype 47S103 which encodes various class of proteins and enzymes, such as Plant actin-related protein 8 (Arp complex) acts as a host response to pathogen infection (Qi et al., 2017), the heavy metal-associated domain plays an important role in the development of vascular plants and in plant responses to environmental changes. Phosphatidylinositol 3-/4-kinase (PI3K) acts as a catalytic domain and plays vital roles in the regulation of various cellular activities, including proliferation, differentiation, membrane ruffling and prevention of apoptosis (Cantley, 2002). Exocyst subunit Exo70 family protein (EXO70) describes the expression profiling of EXO70 genes from wheat (Zhao et al., 2019). Seven candidate genes were found for pathotype 46S119. Out of seven genes, *TraesCS6D02G384800* gene on 6DL was annotated leucine-rich repeat receptor-like protein kinases and serine-threonine/tyrosine-protein kinase (STPK) which has key roles in pathogen recognition and disease resistance. Further, six candidate genes (Table 6) identified against pathotype 110S119 were annotated, and they encoded Isopenicillin N synthase-like, Agent domain plant type, Isopenicillin N synthase-like, bifunctional inhibitor/plant lipid transfer protein, Mitochondrial substrate/solute carrier and leucine-rich repeat receptor-like protein kinases, respectively. The candidate genes annotated for 238S119 revealed functions, such as amino acid pathway regulation, transcription regulation, DNA repair and metabolite transfer.

**Table 6:** Candidate genes identified in significant associated SNPs with studied pathotypes.

**Pathotype**	**Marker**	**Chr**	**Position (Mb)**	**Gene**	**Gene description**
**47S103**	AX-95166280	2DL	621.02	*TraesCS2D02G542500*	Exocyst subunit Exo70 family protein
	AX-95007159	2DL	619.6	*TraesCS2D02G538600*	Sulfotransferase
	AX-94877000	3B-	3.24	*TraesCS3B02G005900*	Transcriptional regulatory protein Sin3-like
	AX-95098367	3DS	3.98	*TraesCS3D02G010600*	integral component of membrane
	AX-94417619	3DS	12.45	*TraesCS3D02G034000*	Proteinase inhibitor I12, Bowman-Birk
**46S119**	AX-94904447	3B-	3.24	*TraesCS3B02G005900*	Transcriptional regulatory protein Sin3-like
	AX-94702815	3B-	542.74	*TraesCS3B02G336700*	integral component of membrane
	AX-94797468	5AS	253.6	*TraesCS5A02G119800*	Inositol monophosphatase-like
	AX-94706157	5BS	38.36	*TraesCS5B02G035500*	FAS1 domain superfamily
	AX-95145565	5BS	45.24	*TraesCS5B02G040800*	Bidirectional sugar transporter SWEET
	AX-94429414	5DS	98.26	*TraesCS5D02G091100*	Amino acid transporter, transmembrane domain
	AX-94859969	6DL	462.86	*TraesCS6D02G384800*	Leucine-rich repeat domain superfamily/Leucine-rich repeat-containing N-terminal, plant-type
**110S119**	AX-94883935	3B-	816.28	*TraesCS3B02G592000*	Isopenicillin N synthase-like
	AX-94989376	6AS	221.88	*TraesCS6A02G186500*	Agenet domain, plant type
	AX-94633926	6DL	369.66	*TraesCS6D02G261300*	Isopenicillin N synthase-like
	AX-94844913	7AS	8.78	*TraesCS7A02G021700*	Leucine-rich repeat domain superfamily/P-loop containing nucleoside triphosphate hydrolase
	AX-94878859	7DS	16.7	*TraesCS7D02G031800*	Bifunctional inhibitor/plant lipid transfer protein
	AX-95227592	7DL	518.05	*TraesCS7D02G400800*	Mitochondrial substrate/solute carrier
**238S119**	AX-94381637	1AL	542.87	*TraesCS1A02G362200*	F-box-like domain superfamily
	AX-95229302	1BL	608.47	*TraesCS1B02G376800*	Phosphoribosylformylglycinamidine synthase PurL
	AX-95258852	5BL	499.12	*TraesCS5B02G316700*	Domain unknown function DUF295
	AX-95202253	5DL	428.83	*TraesCS5D02G340400*	Thioredoxin-like superfamily

**Figure 7:**
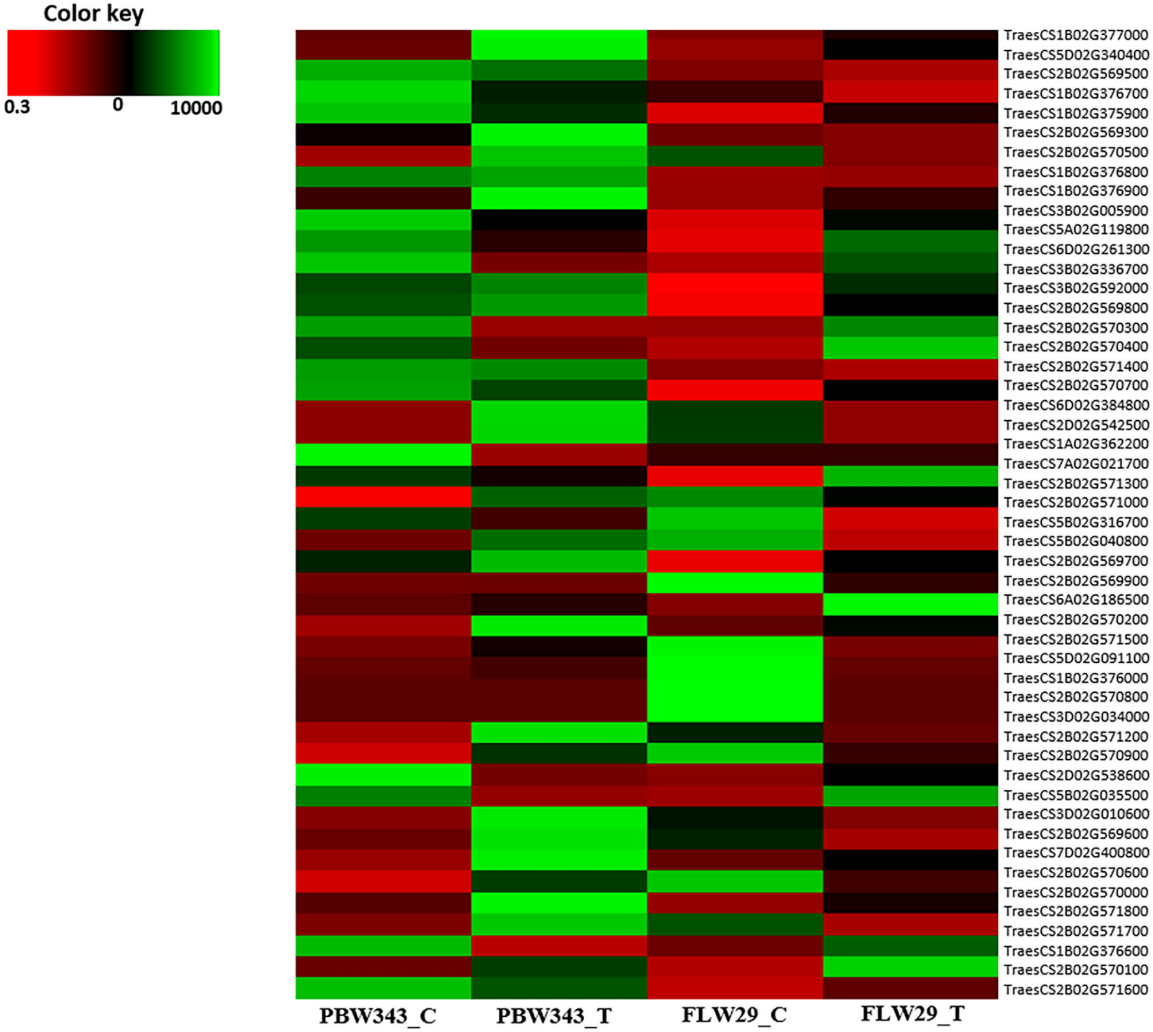
Heat map representing expression of putative candidate genes.

The putative candidate genes were further validated using expression data available in the public domain. The expression of candidate genes was checked against susceptible (PBW343) and resistant (FLW29) wheat cultivars and their expression has been represented through heat map (Figure 7 and Supplementary Table 7).

### Effects of Favorable Alleles on Response to Different Pathotypes

**Figure 8:**
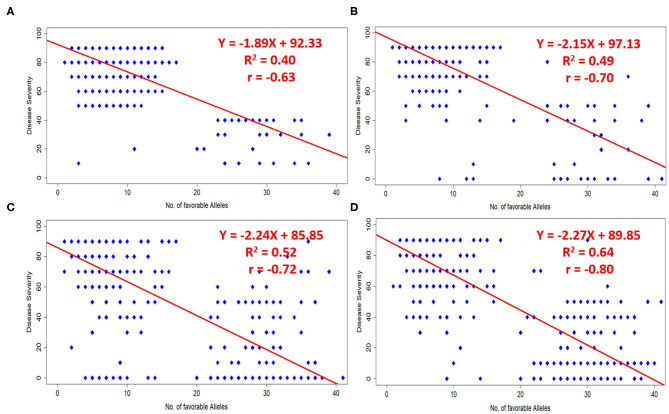
Effects of favorable alleles on response to four pathotypes **(A)** 110S119, **(B)** 238S119, **(C)** 46S119, and **(D)** 47S103.

A total of 51 MTAs (40 QTLs) in relation to different pathotypes were identified. The number of favorable alleles ranged from 1 to 41 for studied germplasm lines. Alleles associated with a reduction in disease response were considered as favorable alleles at each locus of the respective SNP. Broadly we have considered infection type 1–4 as resistant, infection type 5–6 and 7–9 were considered to be moderate and susceptible, respectively. Disease severity can also be understood as percentage of infection (0–9, i.e., on 10 scale). A significant negative correlation (at *p*-value < 0.0001) was observed in case of all four pathotypes, i.e., 110S119 (−0.63), 238S119 (−0.70), 46S119 (−0.72), and 47S103 (−0.80), between the number of favorable alleles in each genotype to their respective disease severity value. The same fact has also been validated by fitting simple linear regression by considering disease severity as response and number of favorable alleles as an independent factor (Figures 8A–D). Model parameters were found to be significant (at *p*-value < 0.0001) with a good range of model R^2^ for all four pathotypes.

## Discussion

### Identification of New Sources of Resistance

The evolution of new virulent races of *P. striiformis* has always been a big challenge before wheat scientists. The new virulent races make the existing ruling varieties susceptible as most of the varieties have race specific resistance that can be overcome easily by new virulent pathogens. Heavy yield losses have been reported due to evolution of new pathotypes (Burdon et al., 2014; Hulbert and Pumphrey, 2014). Hence, there is a need for a constant search for new resistance sources for developing resistant cultivars with durable resistance at regular interval. Global as well as National level efforts have been made to explore new sources of resistance by exploring wheat germplasm collection maintained in gene banks (Gurung et al., 2014; Sehgal et al., 2015; Kumar et al., 2016). Most of the germplasm lines conserved in Gene banks have not been utilized in any breeding program therefore, chances of getting novel sources of resistance is comparatively higher in comparison to breeding lines which are already in use in breeding programs. Further, with the advancement of genotyping technology, these germplasm lines can be effectively characterized and utilized by breeding programs (Cavanagh et al., 2013; Wang et al., 2014). In India, prime focus is to evaluate the wheat lines at the seedling stage for resistance against three new, i.e., 110S119, 238S119, and 47S103 and two already known *Yr9*-virulent pathotypes, i.e., 46S119 and 78S84 (Gangwar et al., 2019).

Germplasm lines including landraces are the primary source of genes and a valuable source of resistance, until now very few landraces have been used in wheat breeding programs (Bajgain et al., 2015; Naruoka et al., 2015; Turner et al., 2017). In our study, significant phenotypic variation among the genotypes has been observed as depicted by ANOVA results. On the basis of IT scores, our results suggested 110S119 and 238S119 were the most virulent pathotypes in 2018 whereas 110S119 was the most virulent in 2019. Recently, a study of diverse spring wheat panel for stripe rust also showed similar results, i.e., 110S119, the most virulent pathotype (Kumar et al., 2020).

We identified novel resistance sources for all the studied pathotypes. Some of them were resistant against two of the pathotypes which includes IC111888, IC290156. In indigenous wheat germplasm lines including landraces for 2 consecutive years. Germplasm lines with higher levels of resistance against prevailing races of stripe rust can be utilized in breeding rust resistant cultivars in the future.

Most of the breeding lines and varieties included in our study showed resistance against the pathotype 47S103 and 46S119 and it could be because these pathotypes evolved in 1991 and 1996, respectively and most of the breeding programs were focusing on these pathotypes only (Bhardwaj et al., 2014). The other two pathotypes 110S119 and 238S119 came into existence during 2014.

### Marker Coverage and Population Structure

Genetic diversity is the basic requirement of any breeding program. The determination of extent of genetic diversity and population structure are the foremost requirement for initiating and utilizing plant genetic resources in breeding programs and also for genetic studies (Atwell et al., 2010). We found the marker density of 19,090 SNPs on different chromosomes and identified that Genome B showed the highest marker density as compared to sub-genome A and D. Previous studies also revealed highest marker density of polymorphic SNPs on the B sub-genome (Kumar et al., 2020).

Population structure is an important factor which influences LD (Flint-Garcia et al., 2003). Our AM panel contains the genotypes from different geographic regions, out of 391 genotypes, 247 were from northern part of India, 86 were from other parts of India like the central zone (Rajasthan, Madhya Pradesh and Gujrat), or Southern part of India (Karnataka, Tamilnadu, Andhra Pradesh, Maharashtra) while 58 genotypes were exotic. Approximately 50% of genotypes were land races/or locally collected germplasm lines. Population structure determined by both STRUCTURE program and PCA based approach suggested four sub-populations (Kumar et al., 2020). A significant level of admixture may be due to the sharing of the germplasm across breeding programs. However, some collection regions have strong enrichment in specific genetic-based sub-populations, like SP 1 contains 76% of the germplasm lines of Indian origin either landraces or breeding lines and released varieties while 24% breeding lines were procured from the CIMMYT Mexico. Out of 102 genotypes from SP 2, only five lines were from CIMMYT Mexico, the rest were local land races (65) and old varieties (32). SP 3 contained only 10 genotypes, of which two were from CIMMYT and eight were from North Indian Origin. SP 4 contains 51 local land races and 22 breeding lines of Indian origin.

LD is one of the most important factors that determine the power of association analysis. We estimated LD for all the chromosomes of three sub-genomes, i.e., A, B, and D. The critical R^2^ values for genome A, B, and D were 0.14, 0.16, and 0.15, respectively. We observed faster decay of LD in the sub-genome A than the other two sub-genomes (B and D). Many other studies have also showed rapid decay of LD in sub-genome A (Chao et al., 2010; Voss-Fels et al., 2015). Further, as in this analysis, the longest LD decay distance was observed for the sub-genome D, the same has been reported in previous studies (Chao et al., 2010; Voss-Fels et al., 2015; Liu et al., 2017b; Maulana et al., 2018; Qaseem et al., 2019). Since the LD decay is influenced by population composition, it thus may vary in different populations, but broadly the sub-genome D generally has a longer LD decay distance as compared to the other two sub-genomes.

Further, although GWAS enables high resolution mapping of traits, often it might also reveal false positive associations if the confounding factors like population structure and genetic-relatedness among genotypes of the association panel are not accounted for. Therefore, in our analysis, a CMLM based method which accounts for both of these factors was used (Wei et al., 2017). The population structure analysis using model based approach, N-J based phylogeny and PCA revealed four sub-populations in the AM panel. However, the clustering pattern observed in our analysis was not explainable on the basis of geographical origin/source of included germplasm lines. One of the possible reasons for this could be the extensive sharing of germplasm within the wheat breeding program of India in the past six decades.

### Comparison of Identified QTLs With Previously Published *Yr* Genes or QTLs

Our study has demonstrated the power of the GWAS approach in uncovering genomic regions associated with stripe rust resistance. In total, 51 MTAs were identified for four studied stripe rust pathotypes. Based on LD decay distance, we classified these 51 MTAs into 40 QTLs (23 suggestive QTLs and 17 significantly associated QTLs) distributed on all the wheat chromosomes except Chr1D and Chr7B. These QTLs further need to be validated before it can be used in any future breeding programs. Previous studies have also identified *Yr* genes/QTLs distributed on almost all the wheat chromosomes as both major and minor genes are known to be responsible for conferring resistance against stripe rust pathogens (Zegeye et al., 2014; Liu et al., 2017b; Muleta et al., 2017a; Ye et al., 2019; xbib80). Zegeye et al. (2014) identified stripe rust resistance QTLs on 1B, 2A, 2B, 3B, 3D, 5A, 5B, 6D, and 7A. Muleta et al. (2017b) identified stripe rust resistance QTLs on 1B, 2A, 2B, 2D, 5B, and 7B. Whereas, Ye et al. (2019) identified 12 QTLs on the long arms of 1B, 3D, 5A, 5B, and 7B and the short arms of chromosomes 1A, 5A, 6A, 6B, and 7A.

Further, we have compared the location of identified QTLs in this study with that of the previous studies. For some of the QTLs, exact comparison across different studies was difficult due to the difference in the number of markers, mapping populations and the genotyping techniques (SSR or SNP) used in these studies. For such QTLs, chromosome arm location (short/long) seems to be a good criterion and was used in our analysis. The details of the previously identified QTLs are provided in Supplementary Table 6. Important novel and previously identified QTLs for stripe rust resistance identified in our analysis are discussed below.

For pathotype 47S103 (T), our analysis identified 2 QTLs (*Qyr.stripe-2BL.1, Qyr.stripe2BL.2*) on the long arm of 2B. Of these, *Qyr.stripe-2BL.1* at 756.7 Mb explained 27.6% of phenotypic variation for disease severity, and coincided to Yr5 on 2BL, close to marker wsnp_Ex_c2153_4043746 (Zegeye et al., 2014). In fact, another stripe rust resistance gene is Yr7 located at 685Mb on 2BL was also very near to *Qyr.stripe*-*2BL.1* identified in our analysis (Zhang et al., 2009). Another QTL on 2B, *Qyr.stripe-2BL.2* QTL was associated with 7 markers (AX-94490490, AX-95175933, AX-94608940, AX-95142963, AX-95238626, AX-94497849, AX-94868242) and located in the interval 759.82-761.99 Mb and was near to SSR marker Xwmc361 that is close to stripe rust resistance QTL Naxos *(QYr.cass-2BL)* (Ren et al., 2012). Muhammad et al. (2020) had also reported two QTLs on 2BL (*QYr.uaf.2BL.1* and *QYr.uaf.2BL.2)* spanning the genomic region containing three important genes *Yr5, Yr43* and *Yr54*. Further, QTL identified on the small arm of 3BS, *Qyr.stripe-3B* (at 3.24 Mb) coincided with *Qyr.ramp-3B.1* which was associated with infection caused by two stripe rust pathotypes Yr-47S103 and Yr-46S119 (Kumar et al., 2020). The QTL on 6BL, *Qyr.stripe-6BL.2* at 408 Mb was close to Xwmc397 and Xwmc105b SSR markers associated with stripe rust resistance (Christiansen et al., 2006).

For pathotype 46S119, 12 QTLs were identified. Among these, six QTLs (*Qyr.stripe-1BL, Qyr.stripe-3AS, Qyr.stripe-4AL, Qyr.stripe-5AS Qyr.stripe-5BS.2, Qyr.stripe-5DS)* were located in the previously located regions and remaining were novel (*Qyr.stripe-3AL, Qyr.stripe-3B.1 Qyr.stripe-3B.2, Qyr.stripe-4DL, Qyr.stripe-5BS.1* and *Qyr.stripe-6DL)*. The QTL, *Qyr.stripe-1BL* identified on 1BL explained 11.8% of phenotypic variance for disease severity and coincided to the genomic region containing *Yr29*, independently identified by Lan et al. (2014) and Muhammad et al. (2020). The long arm of chromosome 1BL, is considered to be rich in *Yr* resistance genes (Muhammad et al., 2020) which was clearly evident from the detection of 3 QTLs in our analysis, one QTL for pathotype 46S119 and two QTLs for pathotype 238S119. The *Qyr.stripe-4AL* at 602.2 Mb explained 12.1% of phenotypic variance and was very near to *Yr51* and *Yr60* (Randhawa et al., 2014; Herrera-Foessel et al., 2015).

For pathotype 238S119, a total of nine genome regions were identified in our analysis. Among these, three QTLs (*Qyr.stripe-1BL.1*, Qyr.stripe-1BL.2 Qyr.stripe-5BL) were located in the previously identified genomic regions for stripe rust resistance and other six were novel (*Qyr.stripe-1AL, Qyr.stripe-4AS, Qyr.stripe-4DL, Qyr.stripe-5BS, Qyr.stripe-5DL.1 and Qyr.stripe-5DL.2). Qyrstripe-1BL* on long arm of 1BL at 326.58 Mb, defined up to 8.2% of phenotypic variance for disease severity and coincided to the genomic region containing *Yr64* and *Yr65* genes (Cheng et al., 2014). Besides this, there were other *Yr* genes, such as *Yr10* and *Yr29* in the vicinity of the identified QTL on Chr1BL (Liu et al., 2008; Lan et al., 2014; Muhammad et al., 2020). Moreover, we have also identified a haplotype block of 3 SNPs (AX-95243592, AX-95229302 and AX-94850928) on 1B in the interval 606–608 Mb. Since these 3 SNPs were in high LD, this region was considered just one locus, QTL *Qyr.stripe-1BL.2*. A previous study on stripe rust resistance in a durum wheat cultivar Wollaroi revealed *Yr29* (Xgwm818, Xgwm259) on 1BL at 670 Mb, which was close to *Qyrstripe-1BL.2* identified in our study (McIntosh et al., 1995, Bansal et al., 2014). The QTL *Qyr.stripe-5BL* was located at 499.12 Mb on chromosome 5BL. This QTL was found to be in the vicinity of QTL *Qyr.sun-5B*, that provided resistance to adult stage stripe resistance (Bariana et al., 2010). Besides the known QTLs, our study has revealed 5 novel QTLs for stripe rust resistance (pathotype 238S119) distributed on 4AS, 4DL, 5BS, and 5DL. These QTLs were at a large distance from the previously identified QTL on these chromosomes.

Similarly, for 110S119, 7 QTLs were identified which included two (*Qyr.stripe-6AS* and *Qyr.stripe-6DL*) that coincided with previously reported genomic regions and five were novel (*Qyr.stripe-3B, Qyr.stripe-7AS, Qyr.stripe-7DL Qyr.stripe-7DS.1*, and *Qyr.stripe-7DS.2*) (Muhammad et al., 2020). Further, all of them explained <10% phenotypic variance suggesting they were minor QTLs and could be important for providing durable resistance.

Identification of favorable alleles for stripe rust resistance is necessary to enhance the cultivars resistance. In the present study, the correlation between stripe rust resistance for different pathotypes and favorable alleles was highly significant and biologically meaningful. In our data, we identified some indigenous lines which were immune (i.e., having 0 disease severity index) to stripe rust resistance. For example, IC111888 (local germplasm) and IC290156 (local germplasm) from NBPGR, New Delhi was found to be immune in 2 races i.e., 238S119 and 46S119; 46S119 and 47S103, respectively. IC111888 and IC290156 had 41 and 37 favorable alleles. It could be concluded that these germplasm lines have relatively few or low identified resistance-associated favorable alleles and therefore showed high disease severity index. This finding will further provide insights for wheat breeders when choosing the diverse parents as a source of rust resistance to breed wheat for the 12 million hectares prone to this disease.

## Conclusion

The results of the present investigation showed the value of our diverse genetic resources conserved in Indian National Gene bank. The germplasm lines/landraces found resistant in the present investigation are valuable sources of resistance and can be used to achieve durable and diverse resistance against stripe rust. In the present study, out of the 40 identified QTLs, 20 QTLs were potentially novel for stripe rust resistance. Further, three putative candidate genes associated with these QTLs are expected to play major roles in marker-assisted wheat breeding for stripe rust resistance in wheat. Further, Genomic regions identified in the present investigations have significant associations with stripe rust resistance in Indian wheat germplasm. However, identified QTLs need to be examined for favorable SNP alleles associated with resistance genes so that they can be used in breeding programs.

## Data Availability Statement

The datasets generated for this study can be found in Zenodo, doi: 10.5281/zenodo.4058520.

## Author Contributions

AKP: investigation, data recording, data analysis, writing—original draft, and writing-review and editing. SundK: conceptualization, data curation, formal analysis, investigation, writing—original draft, and writing-review and editing. AS: conceptualization, investigation, and writing-review and editing. NB: contributed in bioinformatics analysis. DM: contributed in bioinformatics analysis. DC: investigation and data recording. SM and MG: review and editing. SuneK: formal analysis, assisted in writing original draft, review, and editing. OG: phenotypic evaluation of association mapping panel against four different pathotypes of stripe rust. SubK: prepared the inoculum and created artificial epiphytotic conditions for all four pathotypes for 2 years in greenhouse. AG: germplasm curation and reviewing manuscript. SB: phenotypic evaluation of association mapping panel against four different pathotypes of stripe rust. AR and KS: writing—review and editing. All authors contributed to the article and approved the submitted version.

## Conflict of Interest

The authors declare that the research was conducted in the absence of any commercial or financial relationships that could be construed as a potential conflict of interest.

## Supplementary Material

Supplementary Table 1 Details of wheat germplasm lines and distribution of favorable alleles to different pathotypes among 391 wheat germplasm lines used in the present study.

Supplementary Table 2 Disease severity data of 391 germplasm lines against four different pathotypes at seedling stage in 2 consecutive years.

Supplementary Table 3 List of significant MTAs associated to four pathotypes for year 2018.

Supplementary Table 4 List of significant MTAs associated to four pathotypes for year 2019.

Supplementary Table 5 Candidate genes identified within the interval region for chromosome 2BL (759.82–761.99 Mb) and 1BL (606.84–608.53 Mb).

Supplementary Table 6 Comparison of identified QTLs with previously published *Yr* genes or QTLs.

Supplementary Table 7 Expression of putative candidate genes against susceptible and resistant variety.

## Abbreviations

GWAS: Genome Wide Association Study
SNPs: Single Nucleotide Polymorphisms
MAS: Marker Assisted Selection
QTL: Quantitative Trait Locus
LD: Linkage Disequilibrium
QQ: Quantile-Quantile plot
PCA: Principal Component Analysis
GAPIT: Genomic Association and Prediction Integrated Tool
CMLM: Compressed Mixed Linear Model
MTA: Marker Trait Association
FDR: False Discovery Rate
YR: Yellow Rust
IWGSC: International Wheat Genome Sequencing Consortium
GO: Gene Ontology
BLAST: Basic Local Alignment Search Tool
ANOVA: Analysis of variance
REML: Restricted maximum Likelihood.
