# Supplementary material for: Identification of QTLs/Defense Genes Effective at Seedling Stage Against Prevailing Races of Wheat Stripe Rust in India
Source: Front Genet. 2020 Nov 27;11:572975. doi: 10.3389/fgene.2020.572975 (PMC7728992; doi:10.3389/fgene.2020.572975)
Supplement: Supplementary Table 5 — Candidate genes identified within the interval region for chromosome 2BL (759.82–761.99 Mb) and 1BL (606.84–608.53 Mb). [file Table_5.docx]

**Table S5:** Candidate genes identified within the interval region for chromosome 2BL (759.82 Mb – 761.99 Mb) and 1BL (606.84 Mb – 608.53 Mb)

| **Pathotype** | **Chr** | **Gene** | **Pos (Mb)** | **Gene Description** |
| --- | --- | --- | --- | --- |
| **47S103** | 2BL(2.17Mb) | *TraesCS2B02G569300* | 759.82 | Heavy metal-associated domain superfamily |
|  |  | *TraesCS2B02G569500* | 760.11 | Heavy metal-associated domain superfamily |
|  |  | *TraesCS2B02G569600* | 760.15 | Heavy metal-associated domain superfamily |
|  |  | *TraesCS2B02G569700* | 760.24 | Heavy metal-associated domain superfamily |
|  |  | *TraesCS2B02G569800* | 760.26 | Heavy metal-associated domain superfamily |
|  |  | *TraesCS2B02G569900* | 760.82 | F-box-like domain superfamily |
|  |  | *TraesCS2B02G570000* | 760.87 | Plant actin-related protein 8 |
|  |  | *TraesCS2B02G570100* | 760.89 | Ubiquitin-conjugating enzyme |
|  |  | *TraesCS2B02G570200* | 760.9 | Ribosomal protein L10e/L16 superfamily |
|  |  | *TraesCS2B02G570300* | 760.92 | Regulatory protein RecX |
|  |  | *TraesCS2B02G570400* | 760.93 | Cysteine oxygenase/2-aminoethanethiol dioxygenase |
|  |  | *TraesCS2B02G570500* | 760.94 | Phosphatidylinositol 3-/4-kinase, catalytic domain |
|  |  | *TraesCS2B02G570600* | 760.99 | Protein of unknown function DUF393 |
|  |  | *TraesCS2B02G570700* | 761.27 | F-box-like domain superfamily |
|  |  | *TraesCS2B02G570800* | 761.32 | Isopenicillin N synthase-like |
|  |  | *TraesCS2B02G570900* | 761.32 | GA3ox-B3 protein |
|  |  | *TraesCS2B02G571000* | 761.69 | Papain-like cysteine endopeptidase |
|  |  | *TraesCS2B02G571100* | 761.72 | Exocyst subunit Exo70 family protein |
|  |  | *TraesCS2B02G571200* | 761.78 | Exocyst subunit Exo70 family protein |
|  |  | *TraesCS2B02G571300* | 761.84 | Exocyst subunit Exo70 family protein |
|  |  | *TraesCS2B02G571400* | 761.95 | Nucleotide-diphospho-sugar transferases |
|  |  | *TraesCS2B02G571500* | 761.95 | Exocyst subunit Exo70 family protein |
|  |  | *TraesCS2B02G571600* | 761.97 | Exocyst subunit Exo70 family protein |
|  |  | *TraesCS2B02G571700* | 761.98 | Ubiquinol oxidase |
|  |  | *TraesCS2B02G571800* | 761.99 | Exocyst subunit Exo70 family protein |
| **238S119** | 1BL(1.69Mb) | *TraesCS1B02G375900* | 606.84 | Histone H3.2 |
|  |  | *TraesCS1B02G376000* | 606.84 | Serine-threonine/tyrosine-protein kinase, catalytic domain |
|  |  | *TraesCS1B02G376600* | 608.33 | Histone H4 variant TH011 |
|  |  | *TraesCS1B02G376700* | 608.46 | Histone H3 |
|  |  | *TraesCS1B02G376800* | 608.46 | Phosphoribosylformylglycinamidine synthase PurL |
|  |  | *TraesCS1B02G376900* | 608.53 | Mitochondrial carrier domain superfamily |
|  |  | *TraesCS1B02G377000* | 608.53 | Nucleosome assembly protein (NAP) |
