# Supplementary material for: Identification of QTLs/Defense Genes Effective at Seedling Stage Against Prevailing Races of Wheat Stripe Rust in India
Source: Front Genet. 2020 Nov 27;11:572975. doi: 10.3389/fgene.2020.572975 (PMC7728992; doi:10.3389/fgene.2020.572975)
Supplement: Supplementary Table 6 — Comparison of identified QTLs with previously published Yr genes or QTLs. [file Table_6.docx]

**Table S6:** Comparison of identified QTLs with previously published *Yr* genes or QTLs

| **Pathotype** | **QTL** | **Marker interval** | **Reported QTL/genes** | **Reference** |
| --- | --- | --- | --- | --- |
| 47S103 | *Qyr.stripe-1BS* | AX-94839474 | *YrEDWL-1BS.1, YrEDWL-1BS.2* | Liu et al., 2017 |
|  | *Qyr.stripe-2AL* | AX-95174135 | *Qyr.inra_2AL.2_CampReny* | Tehseen et al., 2020 |
|  | *Qyr.stripe-2BL.1* | AX-94848091 | *Wsnp_Ex_c2153/Yr7/Yr5* | Zegeye et al., 2014 |
|  | *Qyr.stripe-2BL.2* | AX-94490490 - 94868242 | *QYr.uaf.2BL.1,QYr.uaf.2BL.2* | Muhammad et al., 2020 |
|  | *Qyr.stripe-3B* | AX-94877000 | *Qyr.ramp-3B.1* | Kumar et al., 2020 |
|  | *Qyr.stripe-4BL* | AX-94853722 | *YrEDWL-3AS* | Liu et al., 2017 |
|  | *Qyr.stripe-6BL.2* | AX-94382687 | *Xwmc397, Xwmc105b* | Christiansen et al., 2006 |
| 46S119 | *Qyr.stripe-1BL* | AX-94482117 | *Qyr.uaf.1BL.3* | Muhammad et al., 2020 |
|  | *Qyr.stripe-3AS* | AX-95193648 | *YrEDWL-3AS* | Liu et al., 2017 |
|  | *Qyr.stripe-3B.1* | AX-94486149 - 94904447 | *Qyr.ramp-3B.1* | Kumar et al., 2020 |
|  | *Qyr.stripe-4AL* | AX-95630255 | *QYr.uaf.4Al* | Muhammad et al., 2020 |
|  | *Qyr.stripe-5AS* | AX-94797468 | *Qyr.ramp-5A.1* | Kumar et al., 2020 |
|  | *Qyr.stripe-5BS.2* | AX-95145565 | *QYr.uaf.5BS* | Muhammad et al., 2020 |
|  | *Qyr.stripe-5DS* | AX-94429414 | *QYr.uaf.5DS* | Muhammad et al., 2020 |
| 110S119 | *Qyr.stripe-6AS* | AX-94989376 | *QYr.uaf.6AS* | Muhammad et al., 2020 |
|  | *Qyr.stripe-6DL* | AX-94633926 | *QYr.uaf.6DL* | Muhammad et al., 2020 |
|  | *Qyr.stripe-7DL* | AX-95227592 | *QYr.uaf.7DL* | Muhammad et al., 2020 |
| 238S119 | *Qyr.stripe-1BL.1* | AX-94717933 | *Yr9/Lr26/Sr31/Pm8* | McIntosh et al.,1995 |
|  | *Qyr.stripe-1BL.2* | AX-95243592 - 94850928 | *Yr29/Xgwm818/Xgw259* | Bansal et al., 2014 |
|  | *Qyr.stripe-5BL* | AX-95258852 | *YrEXP2, QYr.caas-5BL.1_Libellula, QYr-5B_Oligoculm* | Bariana et al., 2010 |
