# Supplementary material for: Identification of QTLs/Defense Genes Effective at Seedling Stage Against Prevailing Races of Wheat Stripe Rust in India
Source: Front Genet. 2020 Nov 27;11:572975. doi: 10.3389/fgene.2020.572975 (PMC7728992; doi:10.3389/fgene.2020.572975)
Supplement: Supplementary Table 7 — Expression of putative candidate genes against susceptible and resistant variety. [file Table_7.docx]

**Table S7:** Expression of putative candidate genes against susceptible and resistant variety

| **Gene** | **PBW343_Control** | **PBW343_Treatment** | **FLW29_Control** | **FLW29_Treatment** |
| --- | --- | --- | --- | --- |
| **TraesCS1B02G377000** | 1457.83 | 1462 | 1550.5 | 2124.67 |
| **TraesCS5D02G340400** | 1557.67 | 1529.83 | 1452.5 | 1892.33 |
| **TraesCS2B02G569500** | 1351 | 1064.33 | 1434 | 1941.83 |
| **TraesCS1B02G376700** | 1327.67 | 1426.67 | 1238.33 | 1918.67 |
| **TraesCS1B02G375900** | 1247.33 | 1377.33 | 1187.5 | 1940 |
| **TraesCS2B02G569300** | 1203.17 | 1430.33 | 1099 | 1945.17 |
| **TraesCS2B02G570500** | 1789.5 | 1552.5 | 1582.33 | 1747.67 |
| **TraesCS1B02G376800** | 1720.17 | 1381 | 1493.5 | 1567.17 |
| **TraesCS1B02G376900** | 1369 | 1238.17 | 1132 | 1280.67 |
| **TraesCS3B02G005900** | 957.67 | 799.83 | 828.33 | 1289.5 |
| **TraesCS5A02G119800** | 611.5 | 773.83 | 1940.67 | 2568.67 |
| **TraesCS6D02G261300** | 2709.67 | 2100.67 | 2086 | 2792.83 |
| **TraesCS3B02G336700** | 2233.67 | 2263.33 | 2011.5 | 2966.83 |
| **TraesCS3B02G592000** | 396.5 | 295 | 176.83 | 290.17 |
| **TraesCS2B02G569800** | 333.83 | 315.67 | 181.5 | 257.17 |
| **TraesCS2B02G570300** | 360.33 | 325.67 | 249.33 | 265.67 |
| **TraesCS2B02G570400** | 236.17 | 228.5 | 140.33 | 254.33 |
| **TraesCS2B02G571400** | 213.33 | 200.83 | 163.17 | 224 |
| **TraesCS2B02G570700** | 359 | 336 | 27.33 | 26.33 |
| **TraesCS6D02G384800** | 492.67 | 641.83 | 181.5 | 260.17 |
| **TraesCS2D02G542500** | 124.83 | 85.17 | 89.83 | 122.5 |
| **TraesCS1A02G362200** | 92.17 | 76.5 | 52.67 | 83 |
| **TraesCS7A02G021700** | 97.33 | 96.33 | 145.5 | 183.83 |
| **TraesCS2B02G571300** | 3.33 | 2.5 | 77.33 | 134.17 |
| **TraesCS2B02G571000** | 62.17 | 43.33 | 43.17 | 36.83 |
| **TraesCS5B02G316700** | 40.17 | 45 | 28.83 | 37.17 |
| **TraesCS5B02G040800** | 23.5 | 54.83 | 71.5 | 66.5 |
| **TraesCS2B02G569700** | 18.67 | 12.17 | 22 | 15.67 |
| **TraesCS2B02G569900** | 14.17 | 12 | 22 | 20.17 |
| **TraesCS6A02G186500** | 13.33 | 12.67 | 8.17 | 16.33 |
| **TraesCS2B02G570200** | 5.33 | 8.17 | 20.83 | 5.5 |
| **TraesCS2B02G571500** | 5.17 | 29.17 | 2.5 | 9.17 |
| **TraesCS5D02G091100** | 0.67 | 4.5 | 2.17 | 9.67 |
| **TraesCS1B02G376000** | 0 | 0 | 1.83 | 0.5 |
| **TraesCS2B02G570800** | 0 | 0 | 1.83 | 0.17 |
| **TraesCS3D02G034000** | 0 | 0 | 1.5 | 0 |
| **TraesCS2B02G571200** | 0 | 0 | 0.33 | 0 |
| **TraesCS2B02G570900** | 0 | 0.33 | 1 | 2 |
| **TraesCS2D02G538600** | 0.33 | 1.33 | 3 | 2 |
| **TraesCS5B02G035500** | 79.17 | 36.33 | 11.67 | 15.33 |
| **TraesCS3D02G010600** | 0.83 | 6.5 | 238.33 | 580.83 |
| **TraesCS2B02G569600** | 400.67 | 366.17 | 481.33 | 593.83 |
| **TraesCS7D02G400800** | 347.67 | 403.17 | 367.33 | 488.83 |
| **TraesCS2B02G570600** | 323.17 | 420 | 597 | 503.67 |
| **TraesCS2B02G570000** | 581.83 | 624.67 | 538.33 | 811.17 |
| **TraesCS2B02G571800** | 409.17 | 363 | 636.83 | 769.33 |
| **TraesCS2B02G571700** | 6112.67 | 5711.17 | 4841.5 | 4500 |
| **TraesCS1B02G376600** | 3466.17 | 4621.5 | 3191 | 4070 |
| **TraesCS2B02G570100** | 10957.33 | 8715.83 | 7960.5 | 10106.33 |
| **TraesCS2B02G571600** | 35.67 | 40.33 | 0.67 | 2 |
